# Tumor Microenvironment-Mediated Immune Profiles Characterized by Distinct Survival Outcome and Immunotherapeutic Efficacy in Breast Cancer

**DOI:** 10.3389/fgene.2022.840348

**Published:** 2022-03-25

**Authors:** Lijun Xu, Yaomin Hu, Wenwen Liu

**Affiliations:** Department of Geratology, Renji Hospital, School of Medicine, Shanghai Jiao Tong University, Shanghai, China

**Keywords:** breast cancer, tumor microenvironment, immunotherapy, immune profiles, TCGA

## Abstract

**Background:** Numerous reports have highlighted that the tumor microenvironment (TME) is closely linked to survival outcome and therapeutic efficacy. However, a comprehensive investigation of the TME feature in breast cancer (BC) has not been performed.

**Methods:** Here, we performed consensus clustering analysis based on TME cell expression profiles to construct TME pattern clusters and TME-related gene signature in BC. GSVA combined with CIBERSORT and ssGSEA algorithms were applied to evaluate the differences in biological pathway and immune cell infiltration level, respectively. The PCA method was employed to construct TME-score to quantify the TME-mediated pattern level in individual BC patients.

**Results:** We determined two distinct TME gene clusters among 3,738 BC samples, which exhibited distinct survival outcome and enriched biological processes. The TME features demonstrated that these two clusters corresponded to the established immune profiles: hot and cold tumor phenotypes, respectively. Based on TME-related signature genes, we constructed the TME-score and stratified BC patients into low and high TME-score groups. Patients with high TME-score exhibited favorable outcome and increased infiltration of immune cells. Further investigation revealed that high TME-score was also related with high expression of immunosuppressive molecules, decreased tumor mutation burden (TMB), and high rate of mutation in significantly mutated genes (SMGs) (e.g., PIK3CA and CDH1).

**Conclusion:** Assessing the TME-mediated pattern level of individual BC patients will assist us in better understanding the responses of BC patients to immunotherapies and directing more effective immunotherapeutic approaches.

## Background

Breast cancer (BC) remains the most frequently diagnosed malignancy and the main cause of cancer-related mortality for global females, and its incidence and fatality rates are 24.2% and 15.0%, respectively (Siegel et al., 2018; Kalimutho et al., 2019). Numerous conventional therapies, including surgery, radio(chemo)therapy, endocrine therapy and targeted therapy, have been applied in clinical practice, resulting in the significant decline of BC death rate (Wang et al., 2019). Recently, immunotherapy based on immune checkpoint inhibitors (ICIs) has been identified as a promising therapeutic regimen for BC patients and could serve as a complement to traditional schemes for treatment of BC (Emens, 2018; Basu et al., 2019). However, a significant portion of BC patients fail to benefit from checkpoint-blocking antibodies and exhibit an increased risk of immune-related adverse events (Wang et al., 2017). Thus, identification of an accurate signature to predict therapeutic responsiveness of immunotherapies is required.

As a complex and continuously evolving entity, the tumor microenvironment (TME) is composed of various cell populations (immune cells, fibroblasts, endothelial cells, etc.) and extracellular constituents (extracellular matrix, growth factors, cytokines, etc.), which surround malignant cells and are supported by the vascular network (Hui and Chen, 2015; Wu and Dai, 2017; Hinshaw and Shevde, 2019). A series of studies have demonstrated that TME not only has profound effects during cancer growth and metastatic progression but also plays a pivotal role in predicting therapeutic efficacy (Deepak et al., 2020). As for BC, tumor-infiltrating lymphocyte, a TME component, has been identified as a biomarker to reflect patients’ clinical outcome and predict the potential benefits from treatment (Stanton and Disis, 2016; Denkert et al., 2018). There have been plenty of studies on evaluation of the clinical significance of TME components by computational methods, such as single-sample gene set enrichment analysis (ssGSEA), which could assess the relative abundance of each cell infiltration in the TME (Ye et al., 2019). However, few studies attempted to discover the role of comprehensive TME feature in prognosis and therapeutic responsiveness in BC.

Here, we incorporated genomic and transcriptomic data of 3,738 BC samples from TCGA, GSE, and METABRIC datasets to comprehensively evaluate the relationship between TME cell infiltration characteristics and survival outcome and therapeutic response. Two TME gene clusters were identified using consensus clustering analysis, which were characterized by cold and hot tumor phenotypes, respectively. Besides, to quantify the pattern level mediated by TME in individual BC patients, we constructed the TME-score using principal component analysis (PCA), which could predict immunotherapies based on ICIs and chemotherapeutics, suggesting that TME performed an essential role in guiding therapies for BC.

## Materials and Methods

### Acquisition and Preprocessing of BC Datasets

Gene-expression profiles and clinical information of BC samples were obtained from publicly available databases: TCGA cBioPortal, and GEO. A total of 3,738 BC samples were incorporated into this research, including those from TCGA-BRCA (N = 1,091), METABRIC (N = 1904), GSE20685 (N = 327) (Kao et al., 2011), GSE20711 (N = 88) (Dedeurwaerder et al., 2011), GSE42568 (N = 104) (Clarke et al., 2013), GSE58812 (N = 107) (Jézéquel et al., 2015), and GSE88770 (Metzger-Filho et al., 2013) (N = 117) (Table 1). As for TCGA dataset, we downloaded FPKM-normalized values and then transformed them into TPM format, which were more similar to microarray data and more comparable between samples (Wagner et al., 2012). The Homo_sapiens.GRCh38.104.chr.gtf from the ENSEMBLE website was used as an annotation file to map ensemble ID to gene symbol. The scale method provided by R “limma” package was applied to normalize gene expression. We discarded genes with low abundance, whose expression value of 0 accounts for >80% of total samples and calculated the average value for duplicate genes. The selection criteria for BC datasets in the GEO database were as follows: 1) datasets with a sample size larger than 80; 2) datasets created based on the GPL570 platform; and 3) datasets with necessary clinical information, especially overall survival (OS) interval and status. The GPL570 annotation file was used to map the probes. We obtained the METABRIC dataset from cBioPortal and then imputed the missing data using R “impute” package. While merging the expression matrix of these 7 BC datasets into one meta-cohort, we applied the “ComBat” algorithm from R “sva” package to achieve the batch-effect removal (Leek et al., 2012). The genomic mutation data obtained from the UCSC Xena database was used for somatic mutation and copy number variation (CNV) analysis.

**TABLE 1:** Baseline characteristics of breast cancer patients in TCGA, METABRIC and GSE datasets.

	TCGA-BRCA	METABRIC	GSE20685	GSE20711	GSE42568	GSE58812	GSE88770
No. of patients	1091	1904	327	88	104	107	117
Age							
≤65	775 (70.7%)	1160 (61.0%)	305 (93.3%)	NA	68 (65.4%)	72 (67.3%)	NA
>65	321 (29.3%)	743 (39.0%)	22 (6.7%)	NA	36 (34.6%)	35 (32.7%)	NA
Gender							
Male	12 (1.1%)	0 (0%)	0 (0%)	NA	NA	NA	0 (0%)
Female	1084 (98.9)	1903 (100%)	327 (100%)	NA	NA	NA	117 (100%)
Stage							
I	183 (17.1%)	474 (24.9%)	NA	NA	NA	NA	NA
II	621 (57.9%)	800 (57.2%)	NA	NA	NA	NA	NA
III	248 (23.1%)	115 (8.2%)	NA	NA	NA	NA	NA
IV	20 (1.9%)	9 (0.6%)	NA	NA	NA	NA	NA
ER status							
Positive	789 (77.4%)	1458 (76.6%)	NA	42 (48.3%)	64 (65.3%)	0 (0%)	106 (90.6%)
Negative	231 (22.6%)	445 (23.4%)	NA	45 (51.7%)	34 (34.7)	107 (100%)	11 (9.4%)
PR status							
Positive	685 (67.4%)	1008 (53.0%)	NA	NA	NA	0 (0%)	79 (68.1%)
Negative	332 (22.6%)	895 (47.0%)	NA	NA	NA	107 (100%)	37 (31.9%)
HER2 status							
Positive	162 (15.8%)	236 (12.4%)	NA	26 (29.5%)	NA	0 (0%)	7 (6.1%)
Negative	865 (84.2%)	1667 (87.6%)	NA	62 (70.5%)	NA	107 (100%)	108 (93.9%)
Survival status							
Alive	947 (86.4%)	1281 (67.3%)	244 (74.6%)	63 (71.6%)	69 (66.3%)	78 (72.9%)	89 (76.1%)
Deceased	149 (13.6%)	622 (32.7%)	83 (25.4%)	25 (28.4%)	35 (33.7%)	29 (27.1%)	28 (23.9%)

### Evaluation of Cell Infiltration in the TME

Based on the expression profile containing 547 reference genes minimally representing each cell type, CIBERSORT could characterize and accurately calculate the proportion of distinct immune cell components from bulk tumor samples using a support vector regression and deconvolution algorithm (Wagner et al., 2012). To discriminate 22 human immune cell phenotypes sensitively and specifically, the LM22 gene signature was applied to quantify T cells, NK cells, B cells, macrophages, DCs, and myeloid subsets. Gene-expression profiles processed by standard annotation files were uploaded to the CIBERSORT website (http://cibersort.stanford.edu/), with the algorithm running based on the LM22 signature and 1,000 permutations. *p*-value <0.05 was considered as the significance criterion.

### Consensus Clustering Analysis of TME Components

Based on the expression profiles of TME-infiltrating cells, we applied the unsupervised clustering method to construct distinct TME pattern clusters and classified patients for subsequent analysis. To determine the optimal number of clusters and guarantee their stability, the consensus clustering algorithm provided by the “ConsensusClusterPlus” package was performed repeatedly for 100 times (Monti et al., 2003). Consensus clustering, as a highly useful technique in cancer research, could detect unknown groups in a dataset based on intrinsic biological features and no external information. Besides, this method could provide quantitative and visual stability evidence derived from repeated subsampling and clustering.

### Identification of Differentially Expressed Genes (DEGs) Between Distinct TME Clusters

Distinct TME pattern clusters have been determined by the above consensus clustering algorithm. Subsequently, to identify DEGs between distinct TME pattern clusters, we applied the empirical Bayesian approach provided by R “limma” package (Ritchie et al., 2015) to estimate gene-expression changes and screened out DEGs using the adjusted *p*-value <0.05 as the significance criterion.

### Gene Set Variation Analysis GSVA and ssGSEA

R “GSVA” package (Hänzelmann et al., 2013) was utilized to perform GSVA enrichment analysis, which could explore the differences in biological processes between distinct pattern clusters mediated by DEGs. The gene set of c2.cp.kegg.v7.4.symbols.gmt obtained from the MSigDB database was utilized as the well-defined signature (Subramanian et al., 2005), and adjusted *p*-value <0.05 was considered as the significance criterion. To investigate the immune infiltration feature between distinct pattern clusters, ssGSEA was employed to quantify the infiltration levels of 23 different types of immune cell (Ren et al., 2021). Based on a Gaussian fitting model and multidimensional scaling, we estimated the bio-similarity of immune cells, calculated the enriched score of each immune cell, and uniformly distributed the normalized score from 0 to 1.

### Construction of the TME-Score

To quantify the TME-mediated pattern level of individual BC patients, we developed a scoring scheme by the following procedures. Firstly, the prognostic analysis of the overlapping DEGs identified between distinct TME pattern clusters was performed by univariate Cox regression analysis and genes with prognostic impact were extracted. Then, feature selection of these genes with prognostic value was analyzed by recursive feature elimination with random forest and the 10-fold cross-validation method provided by the “caret” package. Finally, the expression profile of the determined genes was employed to perform PCA analysis and we extracted principal component 1 and 2 as the signature score. The advantage of this method is focusing on the score on the set with the largest block of well-correlated (or anticorrelated) genes in the set, while down-weighting contributions from genes that do not track with other set members. The TME-score was defined using a formula similar to previous studies (Zhang et al., 2020; Chong et al., 2021): ∑ (PC1i + PC2i), in which i represents the expression of DEGs with prognostic value.

### Quantification of Predictors Related to Immune Response: Immunophenoscore (IPS), the Estimation of Stromal and Immune Cells in Malignant Tumors Using Expression Data (ESTIMATE) and ICIs

As a superior biomarker to predict the response of anti-PD-1 and CTLA-4 therapies, IPS could calculate the determinants of tumor immunogenicity and depict the cancer antigenomes and intra-tumoral immune profiles (Charoentong et al., 2017). This scoring scheme derived from a panel of immune-related genes, which belong to four classes: suppressor cells, effector cells, immunomodulators or checkpoints, and MHC-related molecules. By averaging the samplewise Z scores of the four classes within the respective category, the sum of the weighted averaged Z score was calculated as the IPS. The ESTIMATE algorithm could take advantage of the unique properties of the transcriptional landscape to obtain the tumor cellularity and tumor purity. By using the ESTIMATE algorithm (Yoshihara et al., 2013), we calculated the immune and stromal score to predict the level of infiltrating immune and stromal cells, and these form the basis to infer tumor purity. Tumor tissues with abundant immune cell infiltration represented a higher immune score and a lower level of tumor purity. Immunotherapy-based common immunosuppressive molecules PD-1, PD-L1, and CTLA-4 have achieved tremendous success in clinical practice; novel immune checkpoint proteins such as TIGIT and LAG3 were also strongly recommended to evaluate immune response.

### Statistical Analysis

All data processing was generated in R-4.1.0. For quantitative data, we performed Student’s t-tests and the Wilcoxon rank-sum test to estimate the statistical significance for normally and non-normally distributed variables, respectively. For comparisons of more than two groups, we used one-way analysis and Kruskal–Wallis test as parametric and nonparametric methods, respectively. To analyze the relationship between tumor pattern clusters and prognosis, we applied the R “Survminer” package to perform Kaplan–Meier survival analysis and the Cox proportional hazards model. The surv-cutpoint function provided by R “survival” package was used to divide patients into high and low TME-score and tumor mutation burden (TMB) groups. All statistical *p*-values were two-sided, with a *p*-value <0.05 as statistical difference and adjusted *p*-value calculated using the Benjamini–Hochberg correction.

## Results

### Landscape of Tumor-Infiltrating Immune Cells in BC

**FIGURE 1:**
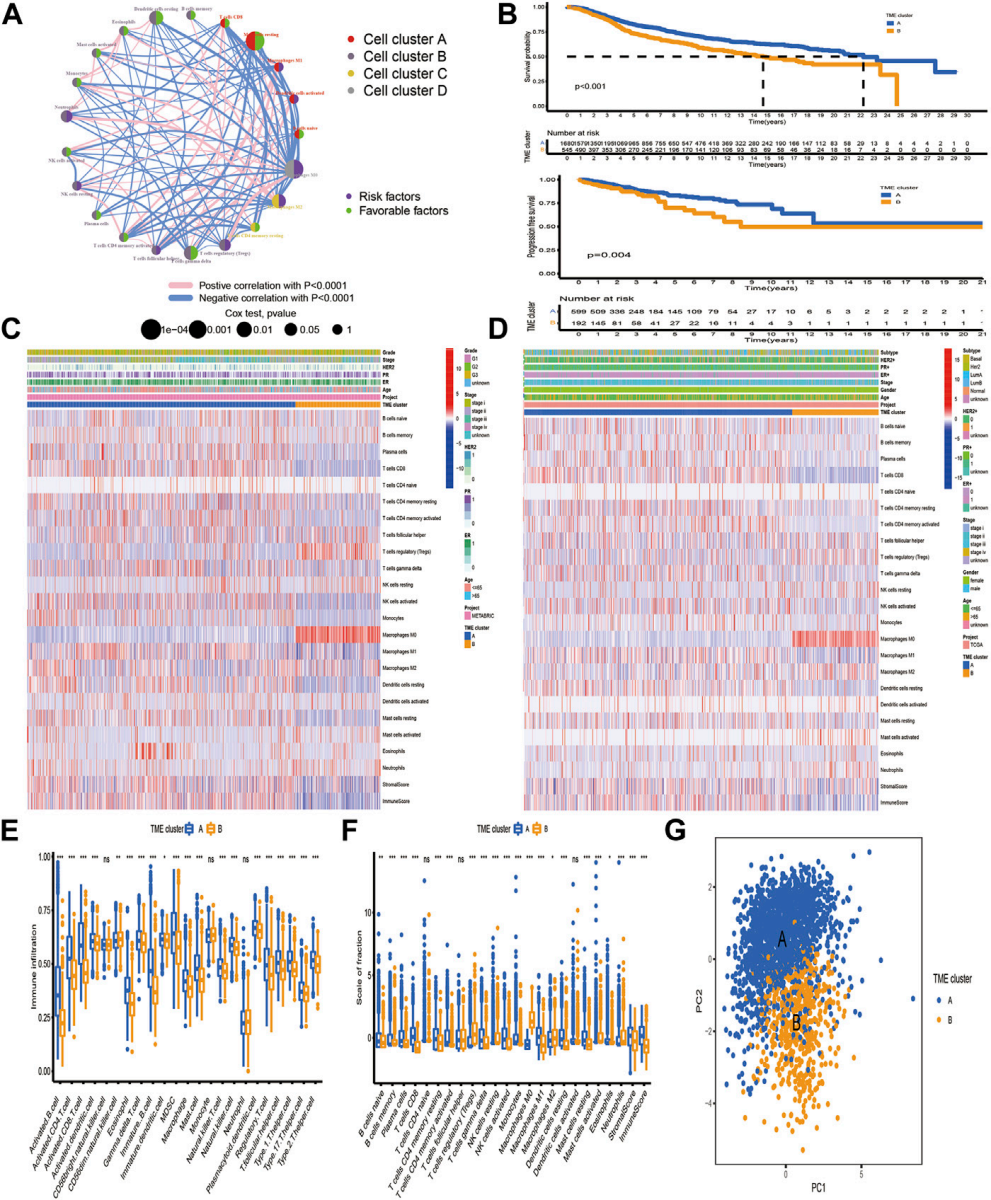
Tumor microenvironment-mediated pattern clusters in breast cancer. **(A)** The interaction between TME-infiltrating immune cells in BC. The lines connecting immune cells represented their interaction with each other. The size of each circle represented the prognostic effect of each immune cell and scaled by *p*-value. Protective factors for patients’ survival were indicated by a green dot in the circle center and risk factors indicated by the purple dot in the circle center. **(B)** Kaplan–Meier curves of OS and PFS for BC patients in the meta-cohort and TCGA-BRCA dataset with distinct TME clusters. The numbers of patients in TME cluster A and B were 1,682 and 545, respectively. **(C,D)** Unsupervised clustering of immune cells in the TCGA-BRCA and METABRIC datasets. Clinicopathological information, including age, gender, ER, PR, HER2, molecular subtype, stage, grade and TME cluster were used as patient annotations. Red represented the high expression of immune cells, and blue represented the low expression of immune cells. **(E, F)** The fraction of tumor-infiltrating lymphocytes in distinct TME clusters using the ssGSEA and CIBERSORT algorithms. Within each group, the scattered dots represented TME cell expression values. The thick line represented the median value. The bottom and top of the boxes were the 25th and 75th percentiles (interquartile range). The statistical difference of two TME clusters was compared through the Kruskal–Wallis H test. *p < 0.05; **p < 0.01; ***p < 0.001. **(G)** PCA of TME-infiltrating immune cells to distinguish TME cluster A from B.

Seven independent BC datasets (TCGA-BRCA, METABRIC, GSE20685, GSE20711, GSE42568, GSE58812, and GSE88770) with completed OS information were merged into one meta-cohort. CIBERSORT was applied using the expression profiles of the meta-cohort to obtain the proportion of 22 immune cells. We classified immune cells into four clusters, and immune cells in each cluster have similar functions. Immune cells in Cluster A recognize antigens and act as messengers between the innate and adaptive immune systems, including activated dendritic cells, resting mast cells, naïve B cells, CD8 T cells, and macrophage M1. Most immune cells in Cluster B have the function of attacking and killing exogenous antigens, such as activated NK cells, T cells regulatory, T cells follicular helper, plasma cells, neutrophils, and activated mast cells. Cluster C includes macrophage M2, which decreases inflammation and encourages tissue repair, and CD4 memory resting T cells. Non-activated macrophage M0 forms Cluster D alone. Univariate Cox regression and Kaplan–Meier analysis were conducted to explore the prognostic impact of immune cells in BC samples, and *p*-value <0.05 was regarded as statistical difference. Figure 1A comprehensively illustrated the landscape of the interactions of the immune cells, cell lineages, and their prognostic impact in BC patients. This network demonstrated that the crosstalk of the infiltration among TME cells significantly correlated with the survival outcome of BC patients and played an indispensable role in the construction of distinct TME pattern clusters. To stratify BC samples with qualitatively different TME-mediated pattern clusters, we performed consensus clustering analysis based on the TME cell expression profiles and determined two distinct clusters, which were termed as TME cluster A, including 1,682 BC samples, and TME cluster B, containing 545 tumor ones (Figure S1). Further analysis of survival outcome revealed that TME cluster A possessed more prominent advantage in OS and progression-free survival (PFS) (Figure 1B).

To further investigate the differences in the biological and clinicopathological characteristics underlying TME cluster A and B, we focused on the TCGA-BRCA and METABRIC datasets, because they compromised the largest BC sample size and the most comprehensive clinical features. As shown in Figures 1C, D, TME cluster A with survival benefit was significantly enriched in macrophages M1 and T cells CD8/CD4 memory activated, while T cells regulatory, macrophages M0, and M2 were mainly concentrated in cluster B. Besides, CIBERSORT and ssGSEA were applied together to estimate the differences in TME profiles between distinct TME clusters, which was highly consistent with the results obtained in the TCGA-BRCA and METABRIC datasets (Figures 1E, F). In terms of clinical features, TME cluster A exhibited a higher stromal and immune score and relatively low tumor purity, which was established by the ESTIMATE algorithm. Based on the TME cell expression profiles, PCA could separate samples with distinct TME clusters into two opposite directions (Figure 1G).

### Identification of TME-Related Signature Genes

**FIGURE 2:**
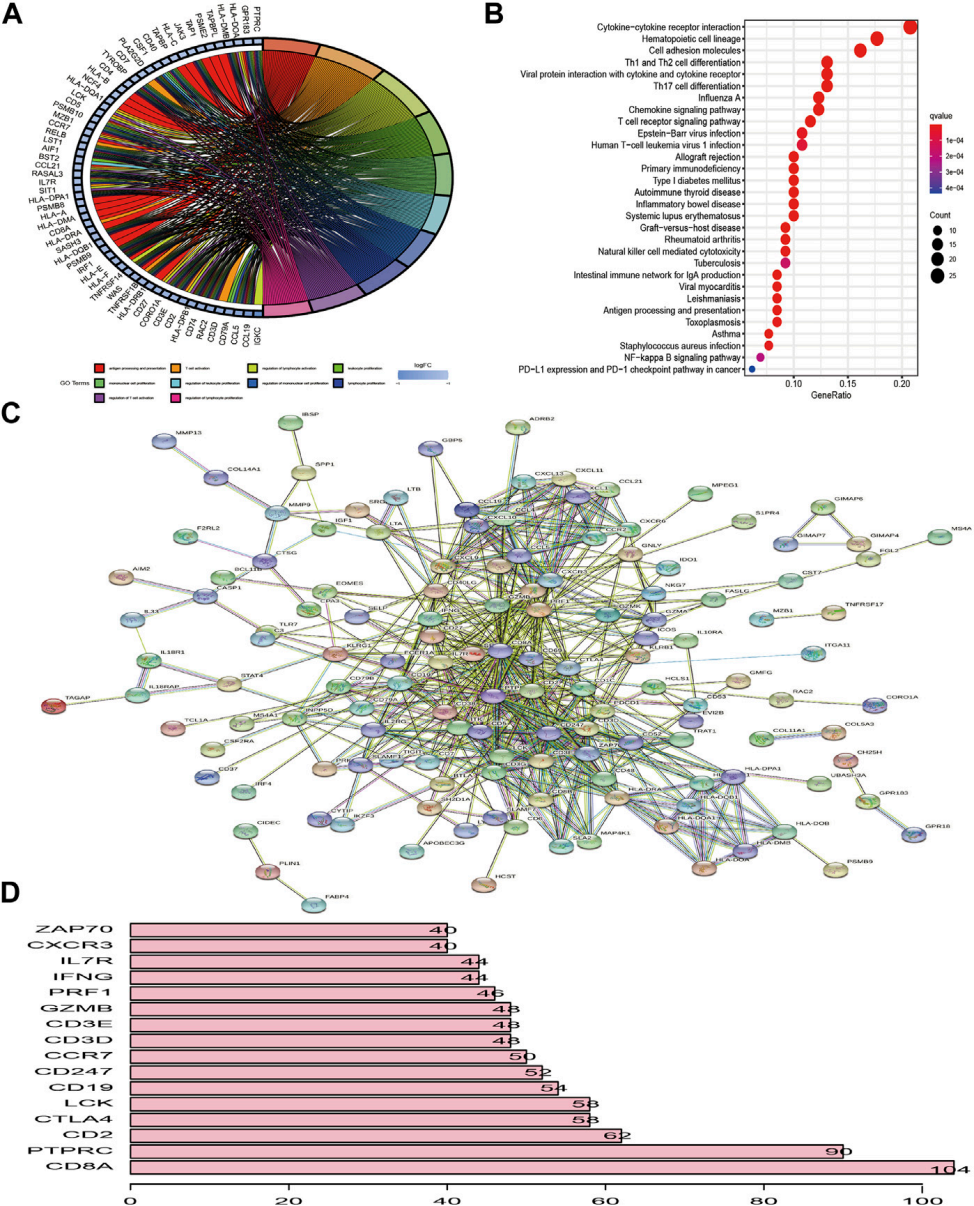
Functional enrichment analysis of differentially expressed genes between distinct TME clusters. **(A, B)** GO and KEGG analysis of DEGs. **(C)** PPI of DEGs. The high confidence (0.700) was considered as the minimum required interaction score. **(D)** Identification of hub genes. Genes with degree of interaction more than 40 were recognized as hub genes. The X-axis label means the degree of interaction of each gene.

Based on TME cell expression profiles, the consensus clustering algorithm could stratify BC patients into 2 distinct TME clusters. However, the genomic alteration and transcriptomic perturbation underlying these 2 clusters were not fully elucidated. Thus, the expression changes of 13,711 genes between the two TME clusters were investigated by the “limma” package and a total of 205 DEGs were determined and regarded as the crucial TME-related gene signature (Supplementary Table S1). Further investigation of the TME-related gene signature demonstrated that these genes were associated with or specific to immune cells, such as BTLA, CD3D, CD3E, CD3G, CD8A, CD8B, CTLA4, ICOS, KLRG1, and PDCD1 for T cells; CD19, CD5, CD79A and CD79B for B cells; and CD48 for dendritic cells. One speculation is that these DEGs were determined between distinct clusters, which were identified based on infiltrating immune cells. Thus, the TME-related signature genes were mainly expressed by various types of immune cell. Then, GO enrichment analysis of these genes demonstrated that biological processes associated with immune activation, such as regulation of lymphocyte proliferation, antigen processing and presentation, and T cell activation, were prominently overrepresented (Figure 2A). KEGG analysis also revealed that these DEGs played an indispensable role in modulating the TME landscape (Figure 2B, Supplementary Table S2). Protein–protein interaction (PPI) was constructed to investigate the interactions among TME-related signature genes, and the high confidence (0.700) was set as the minimum required interaction score. We identified 202 nodes and 587 edges in the PPI network, and 16 genes with degree of interaction more than 40 were recognized as hub genes (Figures 2C, D).

### Landscape of Genetic Alterations of TME-Related Gene Signature in BC

**FIGURE 3:**
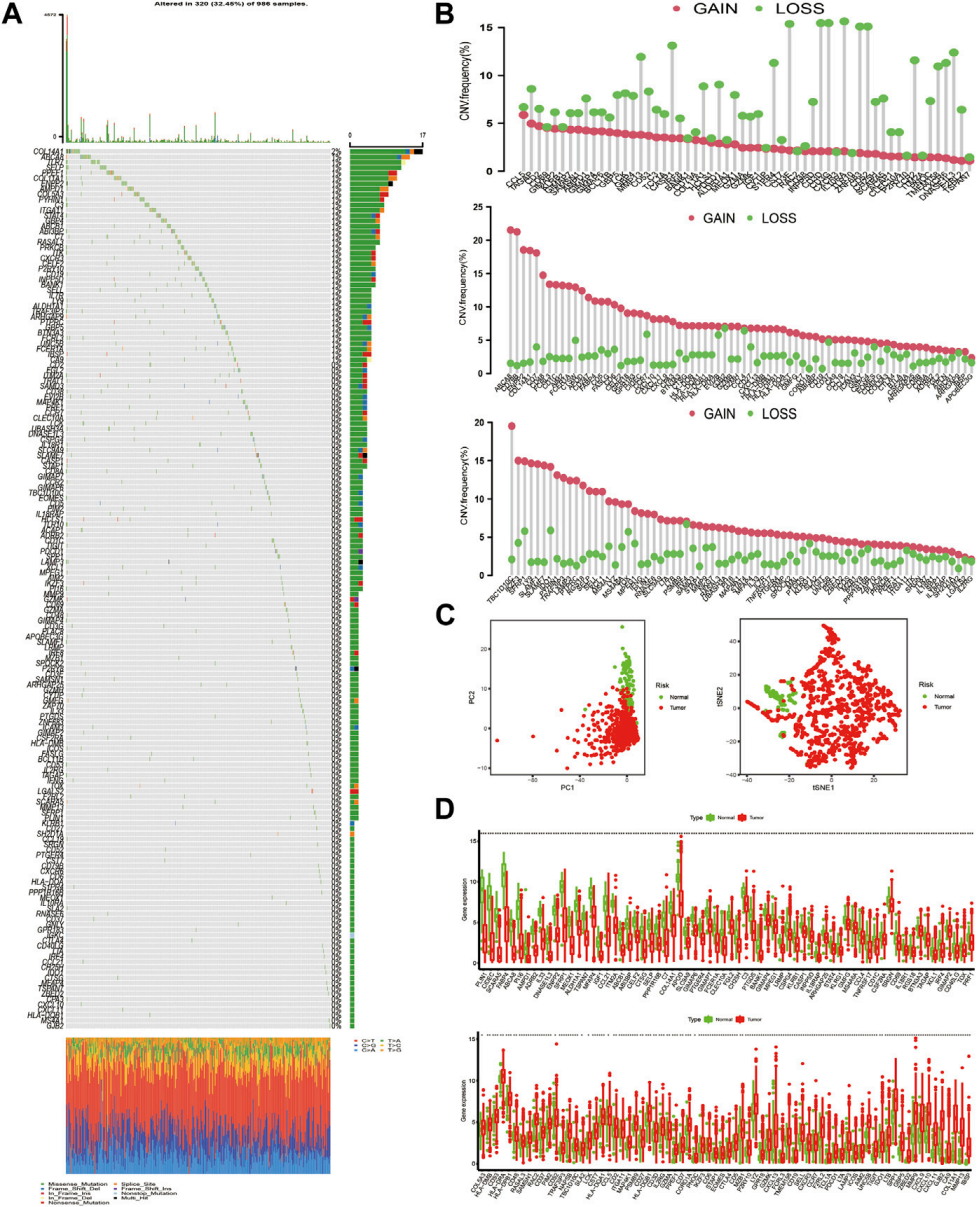
The landscape of genetic alteration of differentially expressed genes in breast cancer. **(A)** 320 of the 986 BC samples experienced genetic alterations, mostly including missense mutation, nonsense mutation, and splice site mutation. The number on the right indicated the mutation frequency of each DEG. Each column represented the individual BC sample. **(B)** The CNV mutation frequency of DEGs was prevalent. The column represented the alteration frequency. The amplification frequency, pink dot; the deletion frequency, green dot. Because of the size of the gene list (205 genes), we presented the CNV results in 3 plots: one for CNV deletion and the other two for amplified CNV. **(C)** PCA and t-SNE analysis based on the expression of DEGs to distinguish BC samples (red dots) from normal ones (green dots). **(D)** The differences in mRNA expression level of DEGs between tumor and normal samples was compared by R “limma” package. The asterisks represented the statistical *p*-value (*p < 0.05; **p < 0.01; ***p < 0.001). For graphical reasons, we presented the gene lists (upregulated and downregulated genes in BC samples) in 2 plots and ordered them based on the log FC.

Figure 3A first depicted the prevalence of somatic mutation of 205 TME-related signature genes among 986 BC samples with available variant classification and variant type information, out of which 320 (32.45%) samples experienced genetic alterations, mainly including splice site mutation, nonsense mutation, and missense mutation. Considering that COL14A1 exhibited the highest mutation frequency, we investigated the difference in the expression of TME-related signature genes between COL14A1-wild and mutant type and a total of 10 genes (ALDH1A1, CH25H, COL11A1, GJB2, HLA-DPB1, IL18R1, MMP13, P2RY10, PTGER4, and UNC5B) were found differentially expressed (Figure S2). Further exploration of 205 DEGs revealed a prevalent CNV alteration. ABCA8, CD79B, TBC1D10C, ENPP2, COL14A1, and CD7 had prevalent CNV amplification, while CD3E, IL10RA, CD3D, CD3G, ZNF683, and CD52 showed widespread CNV deletion (Figure 3B). Interestingly, we discovered that TME-related signature genes exclusively expressed in T cells, such as CD3D, CD3E, and CD3G, exhibited deleted CNV. We speculated that this phenomenon may be attributed to the lower CD3^+^ T cell infiltration. Because distinct TME clusters were determined based on the characteristics of infiltrating immune cells. Besides PCA and t-SNE based on the crucial signature gene expression profiles were performed and BC samples and normal ones could be completely distinguished (Figure 3C). To explore whether the above genetic variation affected transcriptomic expression, we calculated the differences in gene expression value between BC and normal samples and observed that HLA-DRA, HLA-DPB1, and SPP1 were significantly increased in BC tissues, while, FABP4, PLIN1, and APOD were markedly downregulated in tumor tissues (Figure 3D, Supplementary Table S3). As expected, we discovered that the CNV alteration could be the major contributor to perturbation on the expression of the TME-related gene signature. Compared with normal samples, genes with CNV amplification exhibited a remarkedly higher expression in BC samples, such as CD7, IDO1, and LYZ, while other genes with deleted CNV, such as INPP5D, were significantly decreased in tumor tissues (Figures 3B,D), indicating that there was high heterogeneity between the genomic and transcriptomic landscape of TME-related signature genes and this expression imbalance played an essential role in BC tumorigenesis and progression.

### Construction of Pattern Clusters Mediated by the DEGs

**FIGURE 4:**
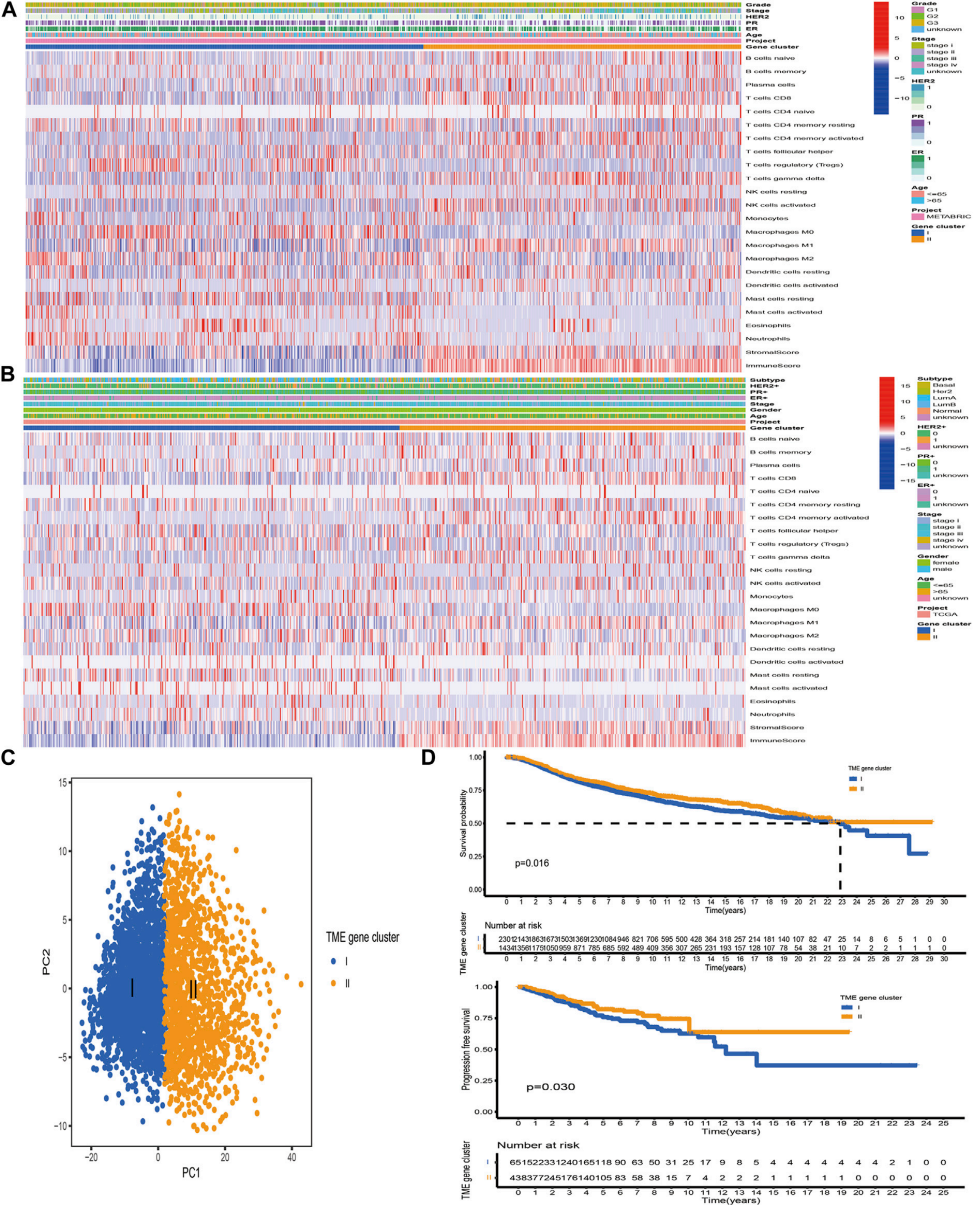
Construction of TME-related gene signature. **(A, B)** Unsupervised clustering of TME-related signature genes in the TCGA and METABRIC datasets. Clinicopathological information including age, gender, ER, PR, HER2, stage, grade, and molecular subtype is shown in annotations above. Red represented the high expression, while blue represented the low expression. **(C)** PCA based on the TME-related gene signature could satisfactorily distinguish between TME gene cluster I and II. **(D)** The Kaplan–Meier curves of OS and PFS for BC samples in the meta-cohort and TCGA-BRCA dataset, respectively.

**FIGURE 5:**
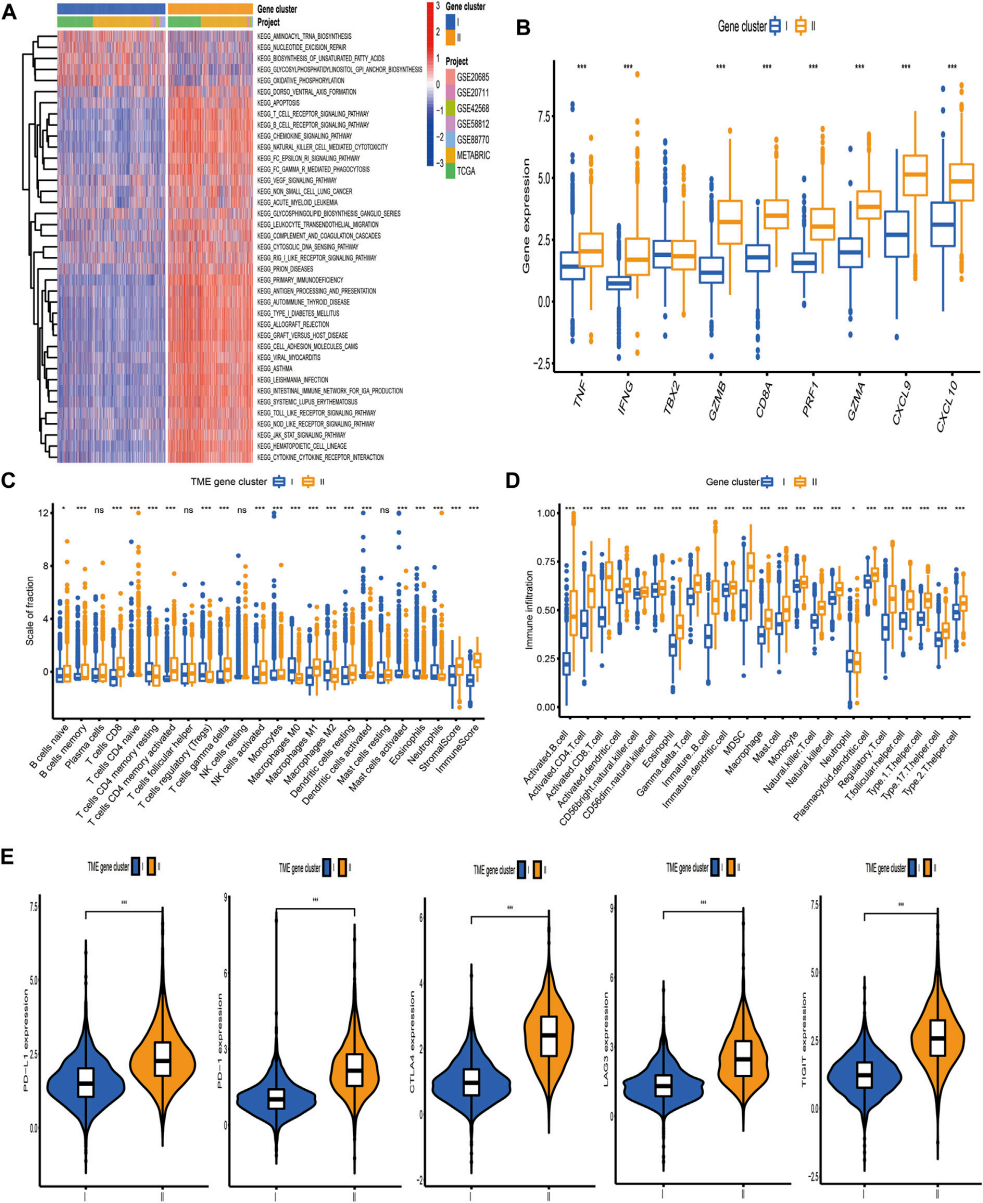
Biological pathways and tumor microenvironment characteristics in distinct gene clusters. **(A)** Heatmap showed the GSVA score of representative hallmark pathways curated from MSigDB between distinct TME gene clusters. **(B)** The differences in mRNA expression of transcripts associated with immune activation between distinct gene clusters. **(C, D)** The fraction of tumor-infiltrating immune cells between distinct gene clusters using the CIBERSORT and ssGSEA algorithms. Within each group, the scattered dots represented TME cell expression values. The thick line represented the median value. The bottom and top of the boxes were the 25th and 75th percentiles (interquartile range), respectively. The statistical difference of the three gene clusters was compared through the Kruskal–Wallis H test. *p < 0.05; **p < 0.01; ***p < 0.001. **(E)** Comparison of the expression level of immunosuppressive molecules between distinct TME gene clusters.

Based on the above hypotheses, we applied consensus clustering algorithm based on the TME-related gene signature to classify BC patients and obtained 2 distinct subtypes, which were defined as TME gene cluster I and II (Figures 4A, B; Supplementary Figure S3). PCA based on the signature gene expression profiles could satisfactorily separate BC samples into opposite directions (Figure 4C). The predictive ability of these 2 gene clusters in survival outcome revealed that gene cluster II exhibited a significant prognostic benefit (Figure 4D). To further explore the biological behaviors between distinct gene clusters, we conducted GSVA and found that cluster II presented enrichment pathways associated with immune activation, including allograft rejection, natural killer cell-mediated cytotoxicity, T/B cell receptor signaling pathway, Toll/NOD-like receptor signaling pathway, antigen processing and presentation, chemokine signaling pathway, and cytokine–cytokine receptor interaction (Figure 5A). Besides, we compared transcripts associated with immune activation between distinct gene clusters and observed a significant upregulation in cluster II (Figure 5B). Further analysis of the TME feature *via* CIBERSORT revealed that compared with gene cluster I, cluster II had elevated infiltration of antitumor immune cells, such as NK cells activated, T cells CD4 memory activated/CD8, and macrophages M1, and a decreased proportion of immunosuppressive or tumor-promoting immune cells, such as macrophages M2 and Treg cells (Figure 5C). Furthermore, we utilized ssGSEA to characterize the immune infiltration profile and observed the similar TME landscape between these 2 gene clusters (Figure 5D). We also applied the ESTIMATE algorithm to evaluate the overall infiltrating level of immune cells and stromal components between these 2 clusters. As expected, we discovered that gene cluster II exhibited an elevated immune and stromal score, indicating that cluster I had a higher tumor purity (Figure 5C). Moreover, some transcripts of immune checkpoint proteins were compared between distinct gene clusters and we observed that PD-L1, PD-1, CTLA4, TIGIT, and LAG-3 were highly expressed in gene cluster II (Figure 5E). Based on these findings, we determined that these 2 TME gene clusters were characterized by distinct immune features. Expectedly, cluster I was defined as cold tumor, characterized by the lack of tumor T cell infiltration and decreased expression of immunosuppressive molecules, and cluster II was recognized as hot tumor, characterized by abundant immune cells and elevated PD-L1 expression.

### Development of the TME-Score and Evaluation of its Clinical Significance

**FIGURE 6:**
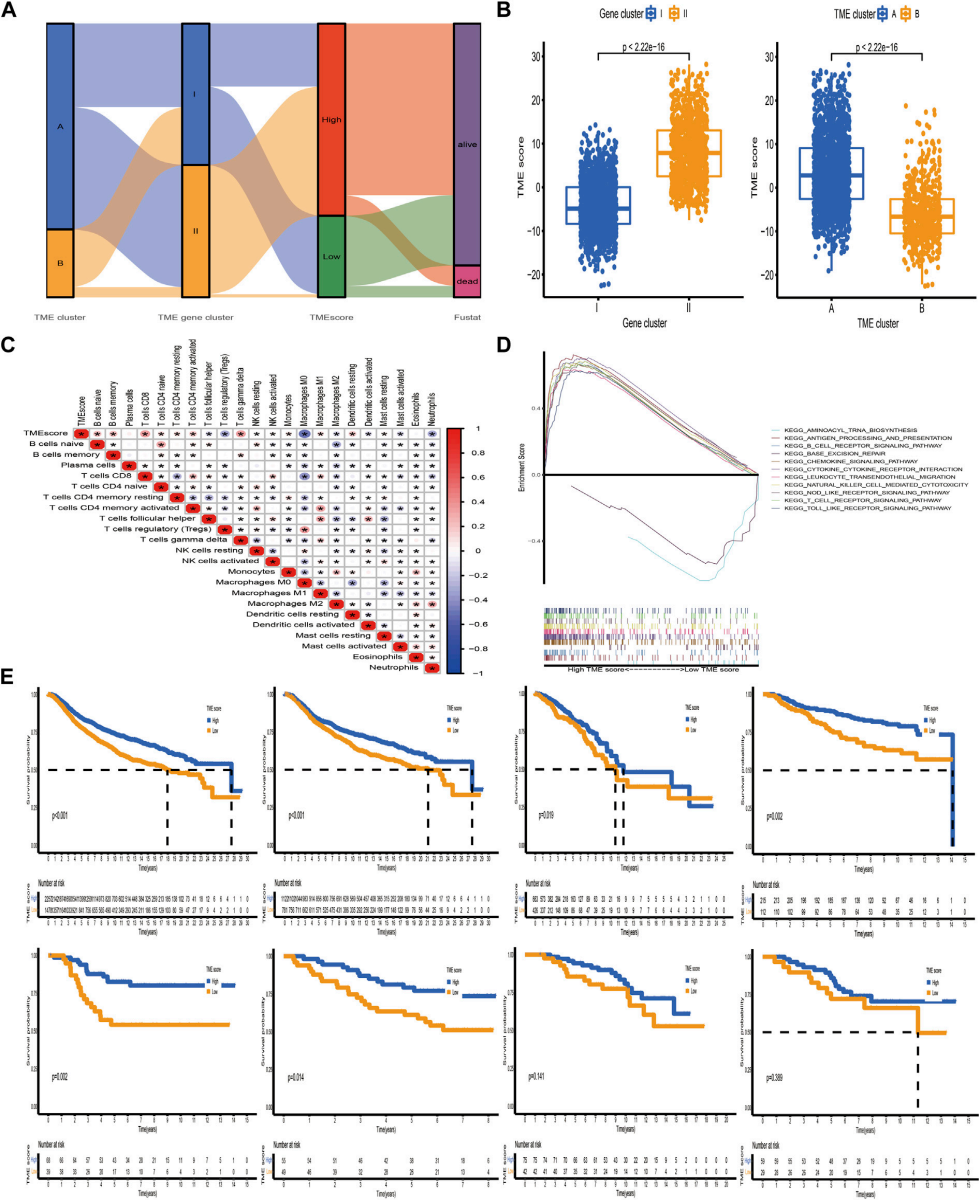
Construction of the TME-score and exploration of its clinical significance. **(A)** Alluvial diagram of the TME cluster, TME gene cluster, and TME-score. **(B)** Relative distribution of TME-score between distinct TME clusters and TME gene clusters. **(C)** Correlations between TME-score and tumor-infiltrating immune cells using Spearman analysis. The asterisks represented the statistical *p*-value (*p < 0.05). **(D)** Representative results of KEGG pathways between high and low TME-score groups *via* GSEA. **(E)** Kaplan–Meier curves for patients with high and low TME-score groups in the meta-cohort and METABRIC, TCGA-BRCA, GSE20685, GSE58812, GSE42568, GSE88770, and GSE20711 datasets. The median TME-score obtained from the METABRIC dataset (discovery cohort) was utilized to separate BC samples into high- and low-score groups. Independent validations were then performed using TCGA-BRCA and GSE datasets (external validation cohort).

The above analyses have demonstrated that TME immune profiles were tightly connected with survival outcome and immune checkpoint proteins in BC. However, these findings were only based on the patient population and could not accurately predict the pattern level mediated by TME in individual tumors. Consequently, we constructed a scoring scheme based on identified TME-related signature genes and defined it as TME-score to quantify the TME-mediated pattern level in individual BC patients. Considering that the quantification of TME-score was complex, we used the alluvial diagram to illustrate the workflow of the TME-score construction (Figure 6A). The Kruskal–Wallis test demonstrated that TME cluster A and gene cluster II with survival advantage exhibited a higher TME-score (Figure 6B). Spearman analysis was performed to examine the relationship between the TME-score and immune landscape. The correlation matrix revealed that the TME-score positively correlated with tumor-infiltrating lymphocytes, including activated CD4+/CD8+ T cells, activated B cells, nature killer T cells, and macrophages M1, and negatively correlated with T cells regulatory, tumor-associated neutrophils, and macrophages M2, demonstrating the crosstalk between TME-score and tumor-infiltrating immune cells (Figure 6C). Moreover, GSEA demonstrated that biological processes associated with immune activation were significantly enriched in the high TME-score group (Figure 6D, Supplementary Table S4). The predictive ability of the TME-score for BC prognosis was estimated by stratifying patients into high- and low-score groups, according to the median value of −3.75 obtained from the METABRIC dataset (discovery cohort). As we expected, patients in the high-score group possessed a prominent survival benefit. Independent validations were performed using the TCGA-BRCA and GEO datasets (external validation cohort) to further assess the prognostic value of TME-score. The median value obtained from the discovery cohort was used to stratify BC patients in the validation cohort into high- and low-score groups. Similarly, patients with a high TME-score exhibited a promising survival outcome, compared with those with a low TME-score (Figure 6E).

### Estimation of the Role of TME-Score in Tumor Somatic Mutation Signature, Immunotherapy, and Chemotherapeutic Efficacy

**FIGURE 7:**
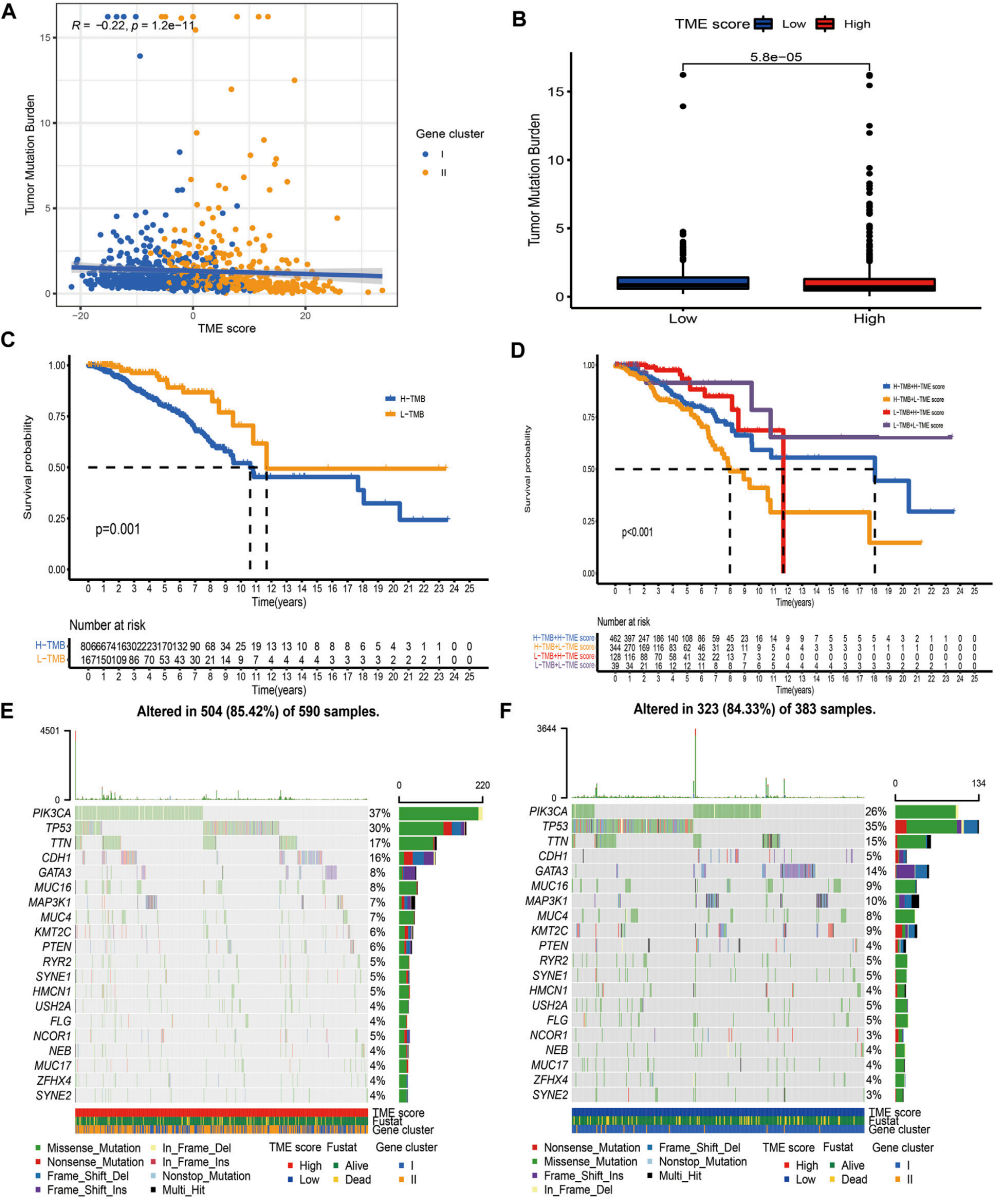
Characteristics of the TME-score in tumor somatic mutation. **(A)** Correlation analysis between TME-score and TMB. **(B)** Relative distribution of TMB in high versus low TME-score groups. **(C)** Kaplan–Meier curve for high and low TMB patient groups (*p* = 0.001). **(D)** Kaplan–Meier curve for subgroup patients stratified by both TME-score and TMB (*p* < 0.001). **(E, F)** Mutational landscape of SMGs in the TCGA-BRCA dataset stratified by high (left panel) versus low TME-score (right panel) groups. Individual patients were represented in each column. The upper bar plot showed TMB, and the right bar plot showed the mutation frequency of each gene in separate TME-score groups.

Accumulating evidence has indicated that cancer-specific antigens produced by somatic mutation could influence the responsiveness to immune-checkpoint blockades. Thus, the distribution pattern of TMB was investigated between high and low TME-score groups. The results demonstrated that TME-score was negatively correlated with TMB and the high-score group exhibited lower TMB (Figures 7A,B). BC patients were categorized into high- or low-TMB group based on the median cutoff value of 0.42. Survival analysis further demonstrated that the high-TMB group had a dismal survival outcome, and as expected, the combined high-TMB and low TME-score group exhibited the worst prognosis (Figures 7C, D). Significantly mutated genes (SMGs) analysis was also conducted between different TME-score groups, and the SMGs landscape revealed that CDH1 (16.27 vs. 4.7%) and PIK3CA (37.29 vs. 26.37%) exhibited higher somatic mutation rates in the high-score group, whereas GATA3 (7.63 vs. 14.1%) had a higher somatic mutation rate in the low-score group (Figures 7E, F; Table 2).

**TABLE 2:** Significantly mutated genes between high and low TME score group.

	High TME-score group		Low TME-score group		*p*-value
	Wild	Mutation	Wild	Mutation	
CDH1	494 (83.73%)	96 (16.27%)	365 (95.3%)	18 (4.7%)	<0.001
PIK3CA	370 (62.71%)	220 (37.29%)	282 (73.63%)	101 (26.37%)	<0.001
GATA3	545 (92.37%)	45 (7.63%)	329 (85.9%)	54 (14.1%)	0.002

**FIGURE 8:**
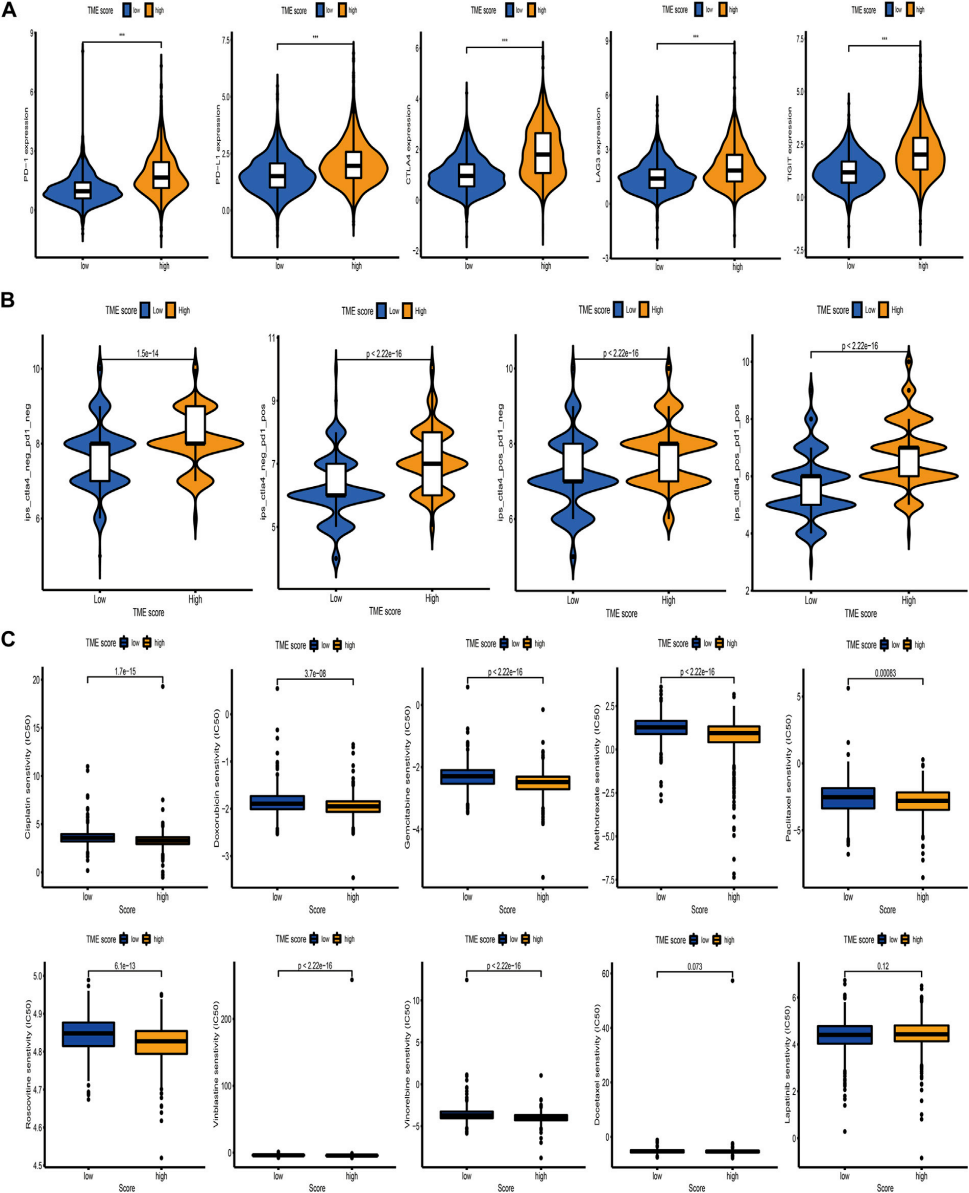
Correlation between the TME-score and immunotherapeutic benefits and chemotherapeutic efficacy. **(A)** The relative distribution of immunosuppressive molecules was compared between TME-score high versus low groups in the meta-cohort. **(B)** Relative distribution of immunotherapeutic efficacy in high TME-score versus low TME-score groups. **(C)** Relative distribution of IC50 for chemotherapeutic drugs commonly used in clinical practice between TME-score high versus low groups.

Immunotherapy based on immunosuppressive molecules has achieved a major breakthrough in antitumor response. Many well-known predictors, especially PD-L1, were extensively used to assess immune response. We discovered that the expression levels of immunosuppressive molecules, including PD-L1, CTLA4, PD-1, TIGIT, and LAG3, were pronouncedly elevated in the high TME-score group (Figure 8A), indirectly demonstrating the essential role of the TME-score in mediating immune response. In addition, we also observed that patients in the high TME-score group exhibited significant therapeutic benefits from ICIs treatment represented by CTLA4-/PD-1-, CTLA4-/PD-1+, CTLA4+/PD-1-, and CTLA4+/PD-1+, indirectly suggesting that the TME-score played an essential role in predicting response to immunotherapies (Figure 8B). In addition to immunotherapies based on ICIs, we attempted to investigate the connection between the TME-score and chemotherapeutic drugs commonly used for BC in clinical practice. We discovered that with the exception of docetaxel and lapatinib, the high TME-score group exhibited decreased IC50 of cisplatin, doxorubicin, gemcitabine, methotrexate, paclitaxel, roscovitine, vinblastine, and vinorelbine, suggesting that the high TME-score group may enjoy therapeutic benefits from these chemotherapeutic drugs (Figure 8C).

## Discussion

Cancer immunotherapies targeting PD-1, PD-L1, and CTLA4 have achieved durable and robust responses for BC patients in clinical practice (Keenan and Tolaney, 2020; Emens, 2021). However, approximately one-third of patients fail to respond to these therapeutic agents, and numerous studies have demonstrated that microsatellite instability, PD-L1/PD-1 expression, and TMB are not efficient predictors of the efficacy of immunotherapies (Zeng et al., 2019; Tang et al., 2020). To maximize the therapeutic benefit, identification of novel predictors for the efficacy of checkpoint-blocking antibodies is essential. Mounting evidence has implied that TME not only takes on an indispensable role in survival outcome but also has profound effects on predicting immunotherapeutic responsiveness (Basu et al., 2019; Emens, 2021). In the present research, we applied several computational methodologies to quantify the infiltration level of immune cells and comprehensively explored the connections between TME-infiltrating immune cells and clinicopathological characteristics, survival outcome, and immunotherapeutic efficacy in BC.

Based on TME cell expression profiles, we employed consensus clustering analysis to determine two TME clusters, which exhibited distinct survival outcome. We speculated that this survival discrepancy may derive from the distinct immune class: TME cluster A was characterized by abundant antitumor T cells CD8 and macrophages M1, corresponding to the immune-activated subtype, while TME cluster B was characterized by immunosuppressive T cells regulatory and higher tumor purity corresponding to the immune-suppressed subtype.

Moreover, DEGs identified between immune-activated and immune-suppressed subtypes were prominently elevated in biological pathways associated with immune activation, demonstrating that these DEGs were regarded as TME-related gene signature. Based on the TME-related signature genes, two transcriptomic clusters were constructed and characterized by different survival outcome, which was in great accord with the results of TME clusters. One possible speculation for this survival difference is that TME gene clusters were characterized by distinct antitumor immunity. TME gene cluster II was characterized by immune activation and abundant antitumor immune cell infiltration, corresponding to hot tumor phenotype, while gene cluster I was characterized by immune suppression and immunosuppressive immune cell infiltration, corresponding to cold tumor phenotype (Duan et al., 2020). Several studies have demonstrated that TME components were tightly connected with aggressive properties of tumor and the likelihood of immune response (Galon and Bruni, 2019; Zeng et al., 2019). Evaluation of the density of CD3 and CD8 lymphocyte populations at the tumor center and margin was found remarkedly correlated with patients’ survival status and immunotherapeutic efficacy (Pagès et al., 2018). We also confirmed that TME gene cluster II was prominently related with antitumor lymphocyte infiltration and a high expression level of immunosuppressive molecules, indicating its potential value in predicting immunotherapeutic benefits.

Furthermore, a scoring scheme named TME-score was established to quantify the TME-mediated pattern level in individual BC patients and direct therapeutic interventions more precisely. As a result, gene cluster II characterized by a hot tumor phenotype showed a high TME-score, while gene cluster I characterized by a cold tumor phenotype exhibited a low TME-score. In addition, we observed that TME-score could serve as a prognostic biomarker in BC. Further analysis demonstrated that TME-score was markedly linked to immunosuppressive molecules and immunotherapy, implying that the TME-score could influence immunotherapeutic efficacy. Based on these findings, we believed that TME-score could be used in clinical practice to identify immune profiles and direct therapeutic strategies.

Assessment of mutated genes capable of driving tumors is one milestone toward cancer detection and therapeutic approach selection. Here, we observed that compared with the low TME-score group, PIK3CA and CDH1 exhibited elevated mutation rates in the high TME-score group, while GATA3 showed augmented mutation rates in the low TME-score group. Recent studies revealed that CDH1 and PIK3CA mutation in genetically modified mice could result in an immune-related subtype for invasive lobular carcinoma of the breast, which was characterized by enhanced immune infiltration and a strong signature for Treg cells and immunosuppressive molecule-based immune checkpoint activation (An et al., 2018). GATA3 is an essential regulator of immune cell function, and its mutation could result in the dysfunction of normal T cells (Usary et al., 2004). These TME-score mediated driver gene mutations remarkedly correlated with immune activity, highlighting the complicated connection between TME and tumor immunogenomic features.

Although, we obtained seven retrospective BC datasets (including a total of 3,738 samples) and performed a comprehensive analysis toward the correlation between TME and prognosis and immunotherapeutic efficacy in BC. Prospective datasets of BC samples were required to verify our results. Besides, it is appropriate to systematically assess the infiltrating immune cells in the tumor core and invasive margin, considering that distinct tumor regions are essential.

## Conclusion

In conclusion, we comprehensively analyzed TME-mediated patterns of 3,738 BC patients based on tumor-infiltrating immune cells and systematically linked this pattern with prognosis and immunotherapeutic efficacy. This integrated analysis demonstrated that assessing the TME-mediated pattern of individual tumor will assist us in better understanding TME characteristics and directing more effective immunotherapeutic approaches.

## Data Availability

The datasets analyzed during the current study are available in the TCGA (https://portal.gdc.cancer.gov/), GEO (https://www.ncbi.nlm.nih.gov/geo/) and cBioPortal (http://www.cbioportal.org/) databases.

## Abbreviations

BC, breast cancer; ICIs, immune checkpoint inhibitors; TME, tumor microenvironment; ssGSEA, single-sample gene set enrichment analysis; PCA, principal component analysis; OS, overall survival; CNV, copy number variation; DEGs, differentially expressed genes; GSVA, gene set variation analysis; IPS, immunophenoscore; ESTIMATE, estimation of stromal and immune cells in malignant tumors using expression data; TMB, tumor mutation burden; PFS, progression-free survival; PPI, protein–protein interaction; SMGs, significantly mutated genes.
