# Supplementary material for: Tumor Microenvironment-Mediated Immune Profiles Characterized by Distinct Survival Outcome and Immunotherapeutic Efficacy in Breast Cancer
Source: Front Genet. 2022 Mar 25;13:840348. doi: 10.3389/fgene.2022.840348 (PMC8992709; doi:10.3389/fgene.2022.840348)
Supplement: Supplementary file 1 [file DataSheet1.docx]

**SUPPLEMENTARY INFORMATION**

**Figure S1-S3**


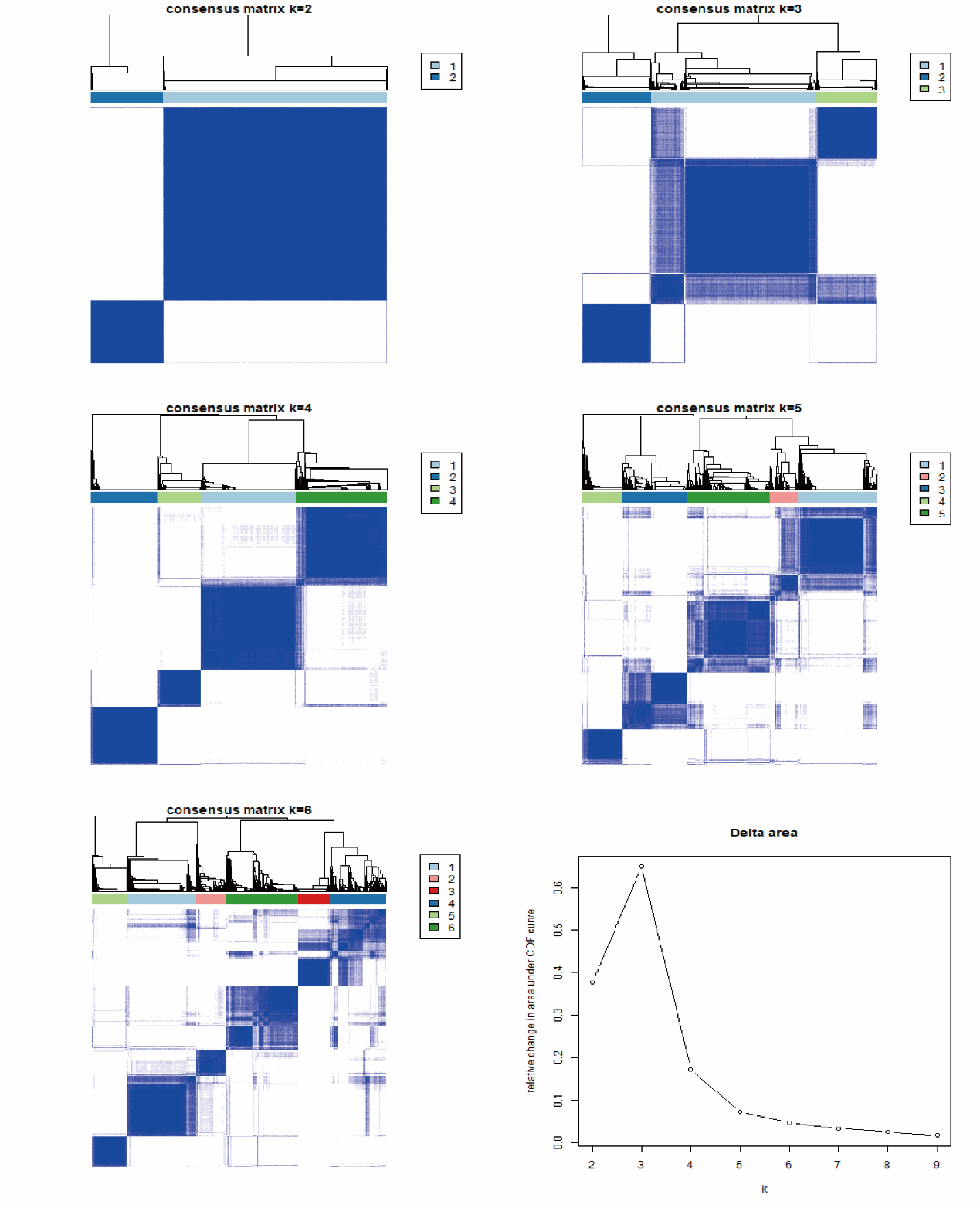


**Figure S1. Unsupervised clustering analysis of tumor microenvironment infiltrating immune cells in one meta-cohort to obtain TME cluster.**

**
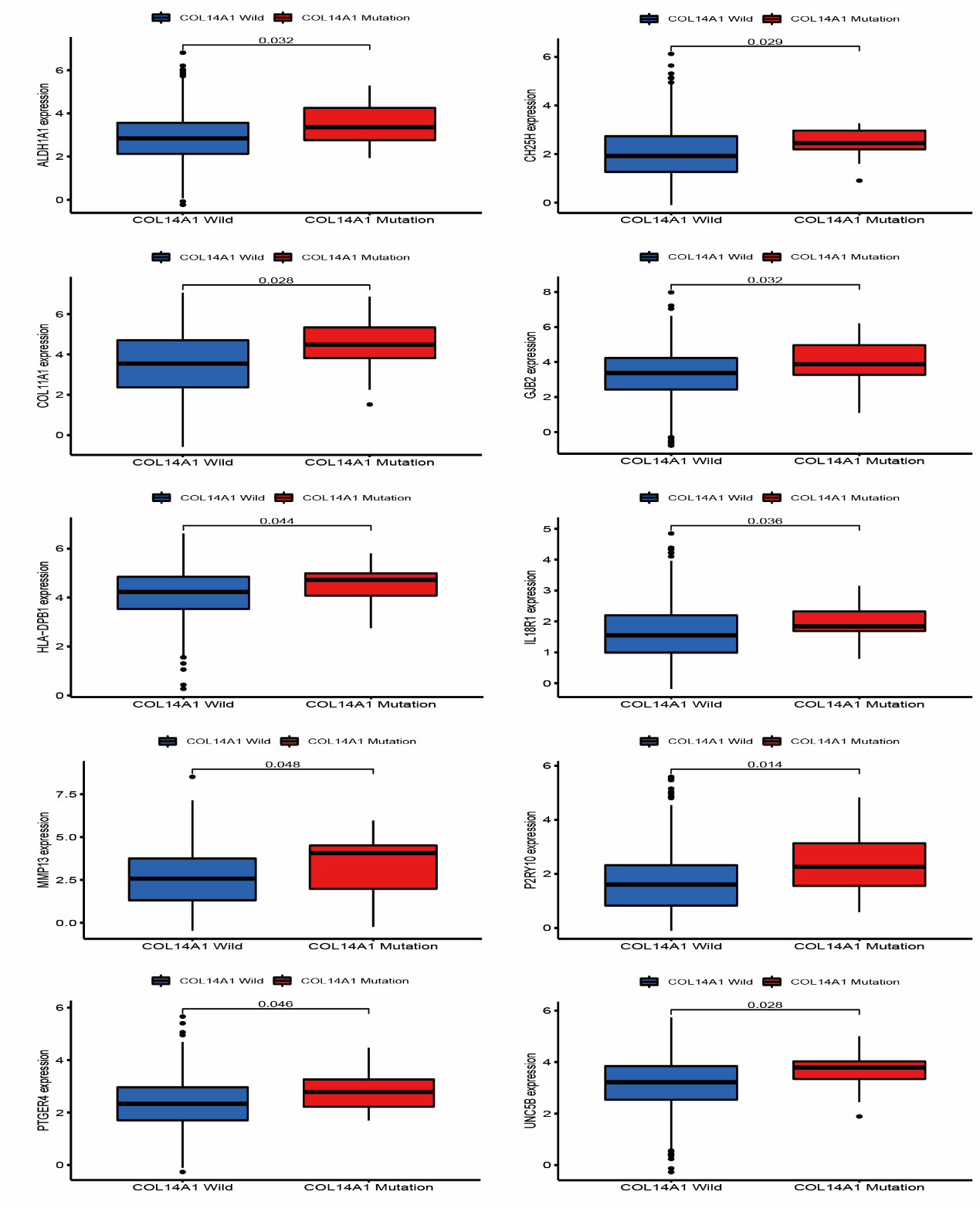
**

**Figure S2. The differences in expression of tumor microenvironment-related gene signature between COL14A1-wild and mutant type.**

**
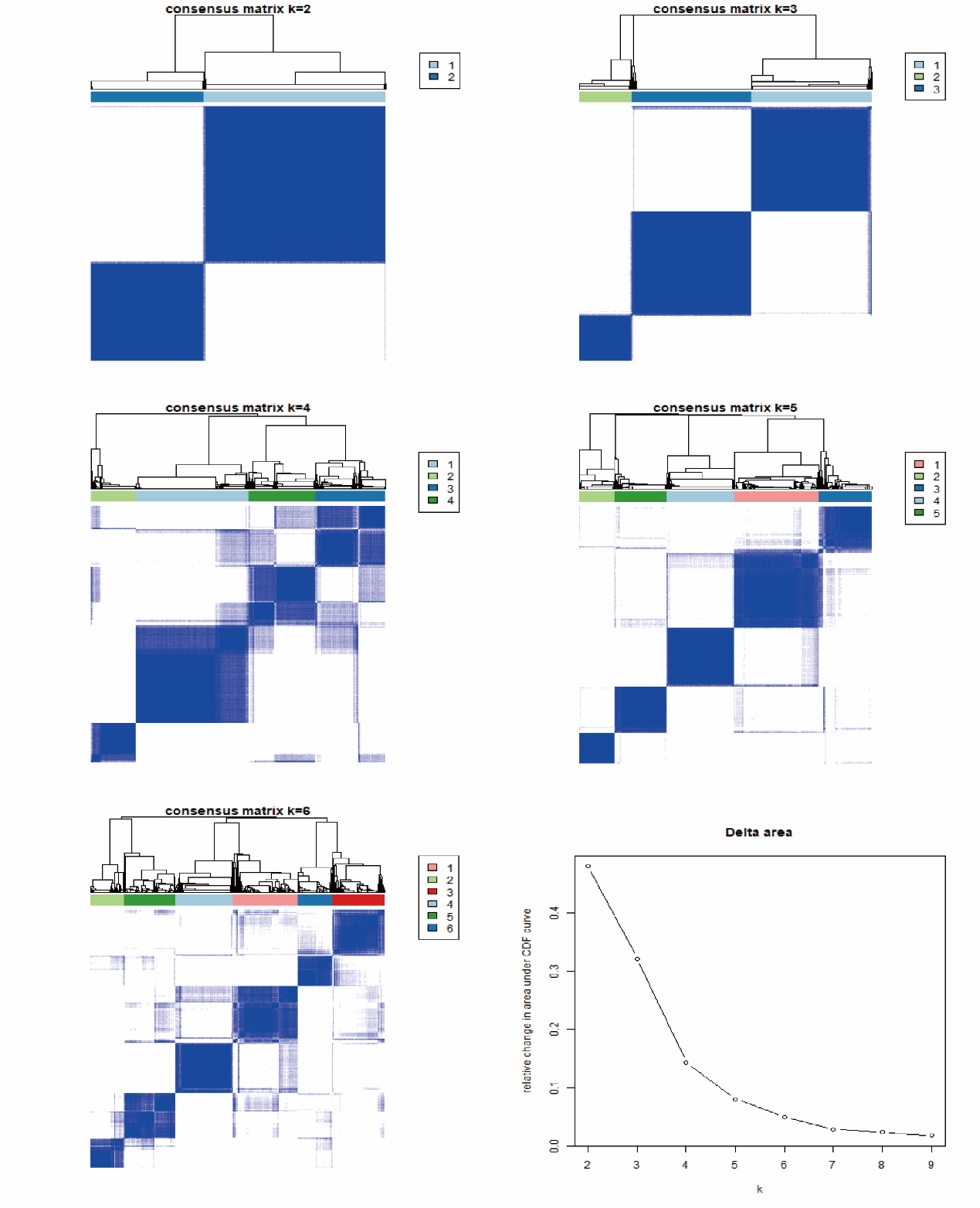
**

**Figure S3. Unsupervised clustering analysis of 2632 tumor microenvironment-related signature genes in one meta cohort to obtain TME gene cluster.**
